# A Kinetic Model of Whole-Body Glucose Metabolism with Reference to the Domestic Dog (*Canis lupus familiaris*)

**DOI:** 10.1155/2015/286076

**Published:** 2015-06-08

**Authors:** Leslie L. McKnight, Anna K. Shoveller, Secundino Lopez, James France

**Affiliations:** ^1^Centre for Nutrition Modelling, Department of Animal & Poultry Science, University of Guelph, 50 Stone Road East, Guelph, ON, Canada N1G 2W1; ^2^The Iams Company, Mason, OH 45040, USA; ^3^Instituto de Ganadería de Montaña (CSIC-ULE), Departamento de Producción Animal, Universidad de León, 24071 León, Spain

## Abstract

A new two-pool model to describe glucose kinetics in the steady state is presented. The pools are plasma glucose, *Q*_1_, and tissue glucose, *Q*_2_ (both *µ*mol). The flows (all *µ*mol/min) into the plasma pool (Pool 1) are absorbed glucose entry from dietary sources, labelled glucose infusion, and hepatic glucose production. There is one flow out of Pool 1, glucose uptake by the tissues. Inflows to the tissues pool (Pool 2) are from plasma and glycogenolysis. Outflows from Pool 2 are to plasma, glucose oxidation, and glycogenesis and other metabolism. Application of the model was illustrated using experimental data derived from healthy adult Labrador Retrievers in the fasted and fed (repeated meal feeding) states. In general, model derived estimates of glucose kinetics were representative of normal glucose metabolism, where rates of glucose production and uptake are similar and act to maintain blood glucose concentrations. Furthermore, estimates of within tissue glucose cycling indicated glycogenolysis in fasting and glycogenesis when fed. In the fasted state, model outputs were consistent with those reported in the canine literature derived using a single pool model.

## 1. Introduction

Diabetes mellitus, a disease characterized by a dysregulation of glucose homeostasis, is increasingly prevalent in companion animals (reviewed by [1]). Accordingly, understanding the regulation of glucose homeostasis is of great interest. Experimentally the use of stable (or radioactive) isotope dilution methodology allows for the quantification of pool sizes and flows between compartments involved in glucose metabolism* in vivo* at the whole body level. Isotopic determination of plasma glucose kinetics has been examined in steady and nonsteady state situations and has been resolved using one-, two-, and three-pool models and noncompartmental schemes (reviewed by [2]). For example, Radziuk et al. [3] proposed a two-pool scheme to describe nonsteady state glucose kinetics, and variants of this model have been applied to canids [4, 5] and felines [6]. However, model solution is computationally complex and requires previous knowledge of parameters, measured either in a separate experiment or prior to initiation of experimental conditions. Likely for these reasons, the single pool model [7] has been used extensively in the literature to describe glucose kinetics. The single pool is representative of plasma glucose and rates of appearance (*R*_*a*_) and disappearance (*R*_*d*_) from the pool are considered the sum of all possible entries and exits into and out of the system. In the steady state, such as fasting, *R*_*a*_ describes hepatic glucose production and *R*_*d*_ denotes whole body tissue uptake (disappearance) based on the assumption that glucose is uniformly distributed in the extracellular fluid space. The single pool model is unable to describe the bidirectional exchange of glucose between plasma and body tissue compartments and the contribution of intracellular glucose recycling. To overcome these limitations and yet preserve the inherent simplicity and mathematical convenience of the single pool model, a new two-pool model is proposed to describe glucose kinetics in the steady state.

The primary aim of this study was to develop a simple two-pool model to describe glucose kinetics in the steady state. The model builds from the existing single pool model by giving explicit representation to the bidirectional exchange of glucose between plasma and tissue compartments and the within tissue cycling of glucose through glycogenesis and subsequent glycogenolysis. The proposed model describes glucose kinetics in the steady state and is solved algebraically. To test its validity, the new model was applied to experimental data derived from our previously published work [8] and an analysis of errors was conducted. Model outputs were then compared to glucose *R*_*a*_ and oxidation values in the published canine literature derived using a single pool model.

## 2. Methods

### 2.1. The Model

**Figure 1:**
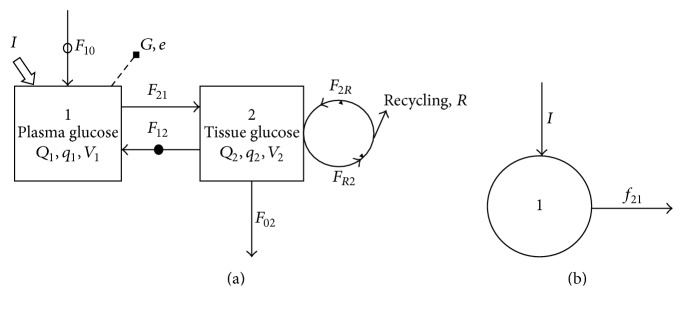
Two-pool model for describing glucose kinetics (a) and the kinetic scheme for labeled glucose (b). The first pool represents total (labelled plus unlabelled) glucose in plasma, and second represents total glucose in tissues. Arrowed solid lines show flows, hollow arrow shows glucose application (infusion), a solid dot indicates a flow active only in the fasted state, a hollow dot indicates a flow active only in the fed state, and the broken line represents sampling. *V*_*i*_ represents the volume of pool *i*, *Q*_*i*_ and *q*_*i*_ represent the quantity of total and labelled glucose in pool *i*, respectively, and *F*_*ij*_ and *f*_*ij*_ represent the flow of total and labelled glucose to pool *i* from pool *j*, respectively.

The kinetic scheme for total glucose (labelled plus unlabelled) comprises two pools and seven flows (Figure 1(a); see Mathematical Notation). The pools are plasma glucose, *Q*_1_, and tissue glucose, *Q*_2_ (both *µ*mol). The flows (all *µ*mol min^−1^) into the plasma pool (Pool 1) are absorbed glucose entry from dietary sources, *F*_10_, labelled glucose infusion, *I*, and hepatic glucose production, *F*_12_. There is one flow out of Pool 1, glucose uptake by the tissues, *F*_21_. Inflows to the tissues pool (Pool 2) are *F*_21_ and glycogenolysis, *F*_2*R*_. Outflows from Pool 2 are *F*_12_, glucose oxidation, *F*_02_, and glycogenesis and other metabolism, *F*_*R*2_. The flow *F*_10_ is assumed to occur only in the fed state and the flow *F*_12_ only in the fasted state. The scheme for labelled glucose assumes no reentry of label into Pool 1 during the infusion period and is shown in Figure 1(b). It contains one pool, labelled plasma glucose (*q*_1_, *µ*mol), one inflow, namely, the rate of infusion *I*, and one outflow, namely, labelled glucose uptake by the tissues *f*_21_. Sampling, for example, total glucose concentration, *G*_1_ (*µ*mol mL^−1^), and enrichment, *e*_1_ (*µ*mol labelled per *µ*mol total glucose), is from the plasma pool.

The fundamental equations are, for total glucose,

(1) dQ1dt=F10+F12+I−F21,dQ2dt=F21+F2R−F02−F12−FR2

and for labelled glucose

(2) $$\begin{array}{c} \frac{dq_{1}}{dt}=I-f_{21}=I-e_{1}F_{21}. \end{array}$$

Assume that steady state is reached after a few hours of infusion such that d*Q*_1_/d*t* = d*q*_1_/d*t* = 0 and d*Q*_2_/d*t* ≈ 0. Therefore,

(3) F10+F12+I−F21=0,F21+F2R−F02−F12−FR2≈0,I−e1F21=0.

This set of 3 simultaneous linear equations can be solved algebraically by hand to give

(4) F21=Ie1,F10+F12=F21−I,F12+FR2−F2R=F21−F02.

When the net flow *F*_*R*2_ − *F*_2*R*_ is positive, glycogenesis (and other metabolism) predominates, and when negative, glycogenolysis predominates. In the fasted state, absorbed glucose entry to the plasma pool from dietary sources is zero and (4) yield

(5) F10=0,F21=Ie1,F12=F21−I,FR2−F2R=F21−F02−F12.

In the fed state, hepatic glucose production is negligible. Equations (4) now yield

(6) F12=0,F21=Ie1,F10=F21−I,FR2−F2R=F21−F02.

The fasted model is therefore given by (5) and the fed model by (6).

### 2.2. Application

The model was applied to data obtained from two separate experiments (A and B) [8]. As the focus of the present study is to develop and describe a novel model of glucose kinetics only the key details of the experimental designs, animals, and methodologies are provided (Table 1). The model was applied to data derived from two steady state situations: fasting and fed. For the fed state, dogs were fed their daily ration divided into equal sized small meals every 25 min. This feeding regimen was based on reported gastric emptying time of ~20 min for dogs fed small meals [9] and was assumed to represent a physiological steady state where circulating glucose concentrations were not changing significantly over time. Furthermore, CO_2_ production has been found to be correlated with labelled carbon recovery in animals fed repeated small meals [10]. Experimentally, glucose oxidation (*F*_02_) was calculated according to [11]:

(7) $$\begin{array}{c} F_{02}=\frac{V_{{\text{CO}}_{2}}\times e_{{\text{CO}}_{2}}}{e_{1}\times C_{\text{corr}}\times 6}, \end{array}$$

where *V*_CO_2__ is the volume of CO_2_ production, *e*_CO_2__ is the isotopic enrichment of expired CO_2_, *e*_1_ is the plasma isotopic enrichment, *C*_corr_ is the bicarbonate correction factor to account for carbon retention, and 6 represents the number of carbon atoms per glucose molecule oxidized.

## 3. Results

Kinetic measurements and calculated flows derived from Experiment A and Experiment B are presented in Table 2 (fasting) and Table 3 (fed), respectively. In Experiment A, plateaus in plasma enrichment were not achieved (due to an over prime of isotope) and steady state enrichments were extrapolated using simple negative exponential curve fitting over the period of postpeak decline. Both measured (last sampling time point) and extrapolated values are given. In fasting, the calculated plasma rate of glucose appearance (*R*_*a*_) (*F*_12_) was similar to its rate of disappearance (*F*_21_). Within tissue glucose recycling (*F*_*R*2_ − *F*_2*R*_) values were negative, suggesting that tissue glycogenolysis was predominant. Calculated flows in Experiment A derived from extrapolated steady state values were higher than those similarly derived from measured values. Indeed, extrapolated steady state values in Experiment A were similar to those observed in Experiment B where isotopic steady state was achieved. During repeated meal feeding, measured glucose oxidation (*F*_02_) and calculated flows (*F*_21_, *F*_*R*2_ − *F*_2*R*_) were greater than those observed in fasting, reflecting the increased availability of exogenous glucose. In agreement, within tissue glucose recycling (*F*_*R*2_ − *F*_2*R*_) values were positive, indicating glycogenesis. Glucose oxidation and calculated flows varied considerably within and between experiments. Specifically, oxidation and calculated flows were greater in Experiment B than Experiment A. In Experiment A, oxidation values were lowest when derived from extrapolated steady state enrichments, whereas calculated flows were lowest when derived from measured enrichments.

### 3.1. Analysis of Errors

A brief analysis was conducted into the effects of measurement errors in infusion rate and plasma enrichment on model solutions using the overall mean of experimentally derived values. The model was solved by perturbing each prescribed variable (i.e., *I* and *e*_1_) in turn by 0, ±10, and ±20%. Each calculated flow (*y*, *μ*mol/min·kg) was then plotted against the perturbation (*x*, %), and a five-point linear regression of *y* on *x* was performed to determine the slope of the line produced. Each average slope was subsequently scaled by its corresponding unperturbed average flow value and multiplied by a hundred to give the scaled slopes dimensions of % change in *y* per % change in *x* (Table 4). In general, measurement error in either input did not substantially impact calculated flows. Overall, fasting plasma flows were most affected by measurement errors in plasma enrichment. It is important to note that measurement errors in plasma enrichment affect not only calculated flows, but also glucose oxidation.

## 4. Discussion

A novel two-pool model to describe glucose kinetics in the steady state was presented. The model is advantageous over the existing single pool steady state model, as it describes the exchange of glucose between plasma and body tissue compartments and estimates within tissue glucose recycling, while preserving the mathematical simplicity of the one pool model. Application of the model to experimental data provided estimates of glucose kinetics that were representative of normal glucose metabolism, where rates of glucose production and uptake are similar and act to maintain blood glucose concentrations. Furthermore, estimates of within tissue glucose cycling indicated glycogenolysis in fasting and glycogenesis in fed states.

In attempt to maintain the inherent mathematical simplicity of the single pool model, certain assumptions were made. For example, in the fed state application of the model, hepatic glucose production was assumed to be completely suppressed and dietary glucose was the sole contributor to plasma *R*_*a*_. However, a nonphysiological dose of exogenous glucose would be required to fully suppress endogenous glucose production. Such a dose would not likely be achieved by intermittently feeding small mixed meals. Therefore, *R*_*a*_ truly reflects glucose entry from exogenous and endogenous sources. Dietary glucose was assumed to enter the peripheral circulation directly. In actuality, ingested glucose is absorbed, released into portal circulation, and taken up by the liver prior to its release into peripheral circulation. Additionally, negligible amounts of ingested glucose serve as a substrate for volatile free fatty acid production in the colon. Moore et al. [12] only accounted for 68% of the labeled glucose administered within a single mixed meal fed to mongrel dogs. Of that, 82% appeared within systemic circulation. These findings suggest that our model calculations might underestimate glucose *R*_*a*_.

In the canine literature, plasma *R*_*a*_ has generally been determined using a single pool model under a wide range of physiological conditions (Table 5). The underlying assumption of the single pool model is that blood glucose is rapidly and uniformly distributed in all physiological pools (i.e., plasma, interstitial, and intracellular). Provided the system is in steady state, such as after an overnight fast, the assumption is considered valid. Mathematically, the minimal model calculates the rate of disappearance from plasma, which is assumed to equal *R*_*a*_. Therefore, literature values of *R*_*a*_ presented in Table 5 were comparable to *F*_21_, glucose uptake by the tissues, in our proposed fasted (and fed) model application. Literature *R*_*a*_ values were similar to those in Experiment A derived from extrapolated steady state values and Experiment B, whereas *R*_*a*_ values derived from measured values in Experiment A were lower than experimental and literature values. These findings indicate that *R*_*a*_ is likely to be underestimated if true steady state enrichment is not achieved experimentally. However, extrapolating steady state enrichments yield reasonable estimates of glucose kinetic parameters and can be employed if necessary.

While fasting glucose kinetics has received much attention, no other studies in canines to our knowledge have assessed fed glucose kinetics in the steady state using the repeated meal feeding technique. Fed steady state kinetics has been mimicked by infusing exogenous insulin above basal concentrations under clamped hyperglycemia (and euglycemia) (see [13] in Table 5). In our model, the plasma *R*_*a*_ from ingested glucose ranged from 35 to 81 *μ*mol/(min·kg) in Experiment A and from 153 to 225 *μ*mol/(min·kg) in Experiment B. In general, these values are comparable to those found by Christopher et al. [13] in dogs under clamped hyperinsulinemia, range 54–168 *μ*mol/(min·kg). However, direct comparisons are not appropriate, given the differences in experimental methodology. Specifically, our experimental data reflect glucose *R*_*a*_ in canines fed intermittent mixed meals, whereas Christopher et al. [13] measured plasma glucose turnover using glycemic clamp methodology. Clamp methodology is useful as it provides a direct estimate of insulin sensitivity. However, a primary advantage of the repeated meal feeding technique is that it allows one to directly examine the effects of dietary ingredients and/or compositions on glucose metabolism in the steady state. Such advantage has particular relevance to animal nutrition as mixed meals contain complex blends of starches and other nutrients, which have differential effects on glucose digestion, absorption, and metabolism. Furthermore, the addition of novel and unconventional dietary protein and carbohydrate sources is becoming increasingly popular in companion animal nutrition.

## 5. Conclusions

In conclusion, a novel two-pool model to describe glucose kinetics in the steady state was presented. The model was tested using experimental data generated from our laboratory and compared to existing literature values. The model yielded fasting plasma glucose *R*_*a*_ values comparable to those reported in the canine literature calculated using the single pool model. While comparable literature values are not available for the fed state, all calculated flows increased above fasting reflecting the addition of exogenous (dietary) glucose. Furthermore, calculated flows were higher in Experiment B where dogs were fed more diet than in Experiment A, suggesting that the fed state application of the model is sensitive to changes in dietary derived glucose supply. Together, these findings support the present model as an improved alternative to the existing steady state single pool model.

## Figures and Tables

**Table 1:** Key experimental details^*∗*^.

Item	Experiment A [8]	Experiment B
Study design	Design: parallel crossover Diets: control (CON) and treatment (TRMT) Duration: 22 d with glucose kinetics assessed on day 19 of study	Design: parallel crossover Diets: control (CON) and treatment (TRMT) Duration: 63 d with glucose kinetics assessed on day 35 of study

Study population	A total of 6 neutered male Labrador Retrievers, 3-black-coated (3.9 yr; 27.3 kg) and 3-chocolate-coated (6.8 yr; 35.0 kg)	A total of 12 black Labrador Retrievers, 5 spayed females and 7 neutered males (27.0 ± 0.6 kg; 4.9 ± 0.2 yr of age)

Key details of glucose kinetics experiment	Isotope: U-^13^C-glucose Prime: 77.8 *μ*mol/kg Constant: 0.136 *μ*mol/min·kg Fasting collections (18 h since last meal): 6 blood samples were taken at 60, 85, 110, 135, 160, and 185 min followed by 4 breath samples taken at 220, 245, 270, and 295 min after isotope infusion (start of isotope infused considered time 0) Postprandial collections: total food ration of 14 g/kg divided into 15 equal sized meals fed in 25 min intervals; 6 blood samples were taken at 60, 85, 110, 135, 160, and 185 min followed by 4 breath samples taken at 220, 245, 270, 295, 320, and 345 min after initial meal	Isotope: U-^13^C-glucose Prime: 8.1 *μ*mol/kg Constant: 0.136 *μ*mol/min·kg Fasting collections (18 h since last meal): 6 fasting blood samples were taken at 90, 105, 120, 135, 150, and 165 min followed by 6 breath samples taken at 180, 205, 230, 255, 280, and 305 min after isotope infusion (start of isotope infused considered time 0) Postprandial collections: total food ration of 23 g/kg divided into 14 equal sized meals fed in 25 min intervals; 6 fasting blood samples were taken at 90, 105, 120, 135, 150, and 165 min followed by 6 breath samples taken at 180, 205, 230, 255, 280, and 305 min after initial meal.

^*∗*^Isotope was delivered by primed constant intravenous infusion, blood samples were collected by jugular venipuncture, and breath was collected via indirect calorimetry.

**Table 2:** Glucose kinetics parameters in fasted adult Labrador Retrievers fed two different diets (control, CON, or treatment, TRMT). Data are expressed as mean and standard error (flows are expressed as *μ*mol/(min·kg)).

Diet	BW, kg	*I*	*e*_1_	*e*_CO_2__	*F*_02_	*F*_10_	*F*_12_	*F*_21_	*F*_*R*2_ − *F*_2*R*_
Experiment A									
CON^*∗*^	30.9 ± 1.6	0.136	0.021 ± 0.001	0.003 ± 0.000	2.8 ± 0.2	0	6.5 ± 0.3	6.9 ± 0.3	−2.5 ± 0.2
TRMT^*∗*^	31.3 ± 1.8	0.136	0.019 ± 0.001	0.002 ± 0.000	2.8 ± 0.5	0	7.3 ± 0.6	7.7 ± 0.6	−2.6 ± 0.5
CON^†^	30.9 ± 1.6	0.136	0.010 ± 0.002	0.005 ± 0.001	2.1 ± 0.2	0	19.2 ± 4.9	20.0 ± 5.1	−1.9 ± 0.2
TRMT^†^	31.3 ± 1.8	0.136	0.011 ± 0.002	0.006 ± 0.001	2.4 ± 0.1	0	14.4 ± 3.4	15.0 ± 3.5	−2.2 ± 0.1
Experiment B									
CON	27.4 ± 0.7	0.136	0.007 ± 0.000	0.001 ± 0.000	3.9 ± 0.4	0	19.7 ± 1.0	19.9 ± 1.2	−3.7 ± 0.5
TRMT	27.6 ± 0.7	0.136	0.007 ± 0.000	0.001 ± 0.000	4.0 ± 0.3	0	19.8 ± 1.2	19.7 ± 0.9	−3.5 ± 0.4

^*∗*^Measured enrichment values (last sampling time point).

^†^Extrapolated steady state enrichments.

**Table 3:** Postprandial glucose kinetics parameters in adult Labrador Retrievers fed two different diets (control, CON, or treatment, TRMT). Data are expressed as mean and standard error (flows are expressed as *μ*mol/(min·kg)).

Diet	BW, kg	*I*	*e*_1_	*e*_CO_2__	*F*_02_	*F*_10_	*F*_12_	*F*_21_	*F*_*R*2_ − *F*_2*R*_	*F*_10_/dose, %^¶^
Experiment A^*∗*^										
CON^†^	30.9 ± 1.6	0.136	0.004 ± 0.000	0.001 ± 0.000	12.5 ± 0.3	34 ± 5	0	35.0 ± 4.7	22 ± 4	16.3 ± 2.2
TRMT^†^	31.3 ± 1.8	0.136	0.005 ± 0.000	0.001 ± 0.000	10.1 ± 1.1	29 ± 2	0	30.5 ± 2.0	20 ± 1	14.1 ± 1.0
CON^‡^	30.9 ± 1.6	0.136	0.003 ± 0.001	0.002 ± 0.001	6.4 ± 0.6	78 ± 34	0	80.6 ± 34.9	72 ± 33	37.5 ± 16.3
TRMT^‡^	31.3 ± 1.8	0.136	0.003 ± 0.001	0.003 ± 0.000	5.7 ± 0.4	48 ± 10	0	49.3 ± 10.6	42 ± 10	22.9 ± 5.0
Experiment B^§^										
CON	27.4 ± 0.7	0.136	0.001 ± 0.000	0.001 ± 0.000	61 ± 18	224 ± 56	0	225 ± 56	163 ± 40	35.6 ± 7.1
TRMT	27.6 ± 0.7	0.136	0.001 ± 0.000	0.001 ± 0.000	37 ± 7	152 ± 30	0	153 ± 30	116 ± 23	35.2 ± 5.7

^*∗*^Dogs were fed 14 g/meal glucose.

^†^Experimental enrichment values (last sampling time point).

^‡^Extrapolated steady state enrichments.

^§^Dogs were fed 23 g/meal glucose.

^¶^Crude estimate of glucose absorption, based on diets providing 760 g glucose/kg fed in 25 min intervals.

**Table 4:** Analysis of measurement errors in infusion rate (*I*) and plasma enrichment (*e*_1_) on model solutions^*∗*^.

Item	*F*_02_	*F*_10_	*F*_12_	*F*_21_	*F*_*R*2_ − *F*_2*R*_
Fasted					
Slope, (Δ*F*)/(Δ*I*)	—	—	0.157 ± 0.036	0.160 ± 0.035	0.001 ± 0.006
Error, %	—	—	1.2	1.2	0.0
Slope (Δ*F*)/(Δ*e*_1_)	−0.062 ± 0.022	—	−0.164 ± 0.037	−0.165 ± 0.036	0.058 ± 0.027
Error, %	3.3		1.2	1.2	3.6
Postprandial					
Slope (Δ*F*)/(Δ*I*)	—	0.873 ± 0.350	—	0.874 ± 0.350	0.874 ± 0.277
Error, %	—	1.0	—	1.0	1.3
Slope (Δ*F*)/(Δ*e*_1_)	−0.303 ± 0.090	−0.905 ± 0.357	—	−0.905 ± 0.357	−0.602 ± 0.295
Error, %	1.0	1.0	—	1.0	1.0

^*∗*^ *I* and *e*_1_ were perturbed in turn by 0, ±10, and ±20%.

**Table 5:** Plasma glucose rate of appearance (*R*_*a*_, *μ*mol/(min·kg)) and glucose oxidation (Ox, *μ*mol/(min·kg)) reported in the canine literature (data means are presented).

Reference	Experimental groups (conditions)	BW, kg	*R*_*a*_	Ox
[14]	2-^3^H [basal^*∗*^; methylprednisolone]	21.1	22; 40.1	—
	3-^3^H [basal; methylprednisolone]	21.1	14.4; 18.4	—
	U-^14^C [basal; methylprednisolone]	21.1	14.4; 19.3	—

[15]	Healthy; obese	12.2; 17.5	15.6; 13.9	—

[13]	Clamped euglycemia [0; 7; 40; 120 mU/kg/h constant insulin infusion]	22	19.1; 17.9; 54.7; 101.7	—
	Clamped hyperglycemia [0; 7; 40; 120 mU/kg/h constant insulin infusion]	22	36; 46.1; 96.1; 167.8	—

[16]	Healthy [saline; AICAR^†^; methypalmoxirate; AICAR and methypalmoxirate]	20.1	13; 17.5; 14.5; 16	—
	Diabetic [saline; AICAR; methypalmoxirate; AICAR and methypalmoxirate]	20.1	32.4; 31.6; 23.6; 21.9	—

[17]	Saline infusion; pulsatile; constant octanoate infusion	—	16.7; 18.3; 14.4	—

[18]	Baseline; 6 wk; 12 wk moderate-fat feeding	27.5; —; 29	14.4; 18.3; 18.3	—

[19]	Saline; nicotinic acid	—	15; 24	6; 14

[20]	Basal	20.4	12.7	—

[21]	Basal [U-^14^C; 3-^3^H]	15.2	8.7; 13.9	—

[22]	Basal	—	24.3	—

[23]	Basal [saline; high FFA^‡^; low FFA infusion]	—	15.3; 15.3; 15.3	5; 4.8; 8.9
	Epinephrine infusion [saline; high FFA; low FFA infusion]	—	15; 15.8; 15.3	4.9; 5.2; 9.7
	Propranolol and phentolamine infusion [saline; high FFA; low FFA infusion]	—	15.2; 15.0; 15.2	5.1; 5.1; 9.8

	Overall mean and SE	20.9 ± 0.6	26.8 ± 4.3	7.1 ± 0.9

^*∗*^Basal refers to fasted rest.

^†^AICAR: 5-aminoimidazole-4-carboxamide ribonucleotide.

^‡^FFA: free fatty acids.

## Mathematical Notation

*F*_*ij*_: Flow of total glucose to pool *i* from pool *j*; *F*_*i*0_ represents external flow of glucose into pool *i*; *F*_0*i*_ represents flow of glucose from pool *i* out of the system (*μ*mol/min)
*f*_*ij*_: Flow of labelled glucose to pool *i* from pool *j* (*μ*mol/min)
*G*: Total glucose concentration in primary pool (*μ*mol/mL)
*I*: Constant rate of labelled glucose infusion into primary pool (*μ*mol/min)
*Q*_*i*_: Quantity of total glucose in pool *i* (*μ*mol)
*q*_*i*_: Quantity of labelled glucose in pool *i* (*μ*mol)
*e*_*i*_: Enrichment of pool *i* (*μ*mol labelled glucose/*μ*mol total glucose)
*t*: Time since isotope first administered (min)
*V*_*i*_: Volume of pool *i*; *V*_1_ represents primary (plasma) pool volume (mL).
